# Identification of Novel Reassortant Shuni Virus Strain in Clinical Cases of Israeli Ruminants, 2020–2021

**DOI:** 10.3390/tropicalmed7100297

**Published:** 2022-10-13

**Authors:** Natalia Golender, Joseph Seffi Varsano, Tomer Nissimyan, Eitan Tiomkin

**Affiliations:** 1 Department of Virology, Kimron Veterinary Institute, Bet Dagan 5025000, Israel; 2 Indi-Vet Ltd., Ashdod 7764933, Israel; 3 Public Health Expert Ltd., Meishar 7685000, Israel; 4 Hachaklait Veterinary Services, Caesarea 3088900, Israel

**Keywords:** simbuviruses, *Orthobunyavirus*, *Peribunyaviridae*, neurological signs, cattle, sheep, goat, abortions, phylogeny, sequencing

## Abstract

The Shuni virus (SHUV) causes an endemic viral infection in Israel and South Africa. It belongs to the Simbu serogroup within the order *Bunyavirales*, family *Peribunyaviridae*, genus *Orthobunyavirus*. Recently, it has been identified in aborted cases of domestic ruminants, young cattle and horses manifesting neural signs and acute death, symptomatic cows, and in carcasses of wild animals. Moreover, SHUV was isolated and identified in humans. In this study, we describe clinical cases of SHUV infection in Israeli domestic ruminants in 2020–2021, which represented clinical manifestations of simbuviral infection including abortions, a neural lethal case in a fattening calf, and an acute symptomatic case in a beef cow. In all cases, SHUV was confirmed by complete or partial viral genome sequencing. There is a significant difference of M and L segments of the novel strains compared with those of all known SHUV strains, while the S segments have more than 99% nucleotide (nt) identity with Israeli and African “Israeli-like” strains previously circulated in 2014–2019. This indicates a reassortment origin of the strain. At the same time, M and S segment nt sequences showed about 98–99% nt identity with some South African strains collected in 2016–2018. Nevertheless, the viral origin and the geographical place of the reassortment stayed unknown.

## 1. Introduction

The Shuni virus (SHUV) is a member of the Simbu serogroup within the order *Bunyavirales*, family *Peribunyaviridae*, and genus *Orthobunyavirus* [1]. In general, orthobunyaviruses are negative-sense RNA viruses, mostly transmitted by mosquitoes or culicoid flies [2]. The genome of orthobunyaviruses comprises three unique segments of single-stranded RNA. The average size of each genome segment of orthobunyaviruses is about 6.9 kb for the large (L) segment, 4.5 kb for the medium (M) segment, and 1.0 kb for the small (S) segment [2]. The S segment encodes the nucleoprotein (N protein) and the non-structural protein (NSs), the M segment encodes a polyprotein precursor that yields the two external glycoproteins Gn and Gc, and the L segment encodes the RNA-dependent RNA polymerase (RdRp) [2,3].

Simbuviruses have worldwide distribution. Akabane (AKAV), Schmallenberg (SBV), and SHUV viruses are the important representatives of the Simbu serogroup, having veterinary significance. In domestic ruminants, three different types of clinical manifestation of the disease caused by these viruses were characterized. The most known clinical manifestations are pregnancy abnormalities and severe fetal malformation summarized under the term “arthrogryposis–hydranencephaly syndrome” [1,4,5]. Other clinical manifestations of the disease, which were reported in adult animals, are mild unspecific clinical signs including fever, diarrhea, or decreased milk yield [4,6]. The last and the rarest clinical manifestation of the disease caused by some strains of AKAV or SHUV might occasionally induce encephalitis in young cattle and horses [7,8,9,10].

Simbuviruses, as representative viruses with a segmented genome, may reassort their genome segments, producing a novel type of virus with unknown features, when two different but closely related viruses simultaneously infect the same cell. Reassortment of simbuviruses has been seen naturally [11,12,13,14] and was also produced artificially in laboratory conditions [15].

SHUV, which was first isolated in the 1960s in Nigeria [16], initially was thought to be non-pathogenic to livestock. However, in 2009, SHUV was found in South African horses that suffered from neurological disease [10], increasing its veterinary significance. In 2014, SHUV was also found in Israel in aborted bovine, ovine, and caprine fetuses [17], further increasing its significance as an important pathogen for livestock. Due to the frequent reports of SHUV detection and isolation in South Africa and Israel, we can presume that SHUV is endemic in these countries [18,19]. Notably, Israeli SHUV strains with a date of detection during 2014–2019 were very closely genetically related, showing only genetic drift [19].

In addition to being identified in ruminants, SHUV was isolated from a febrile child in Nigeria in 1966 [16,20]. Recent research conducted in 2017 in South Africa, in which cerebrospinal fluid samples collected from human hospital patient manifesting neurological symptoms with unknown etiology were examined, showed that 5.4% of tested samples were SHUV positive in RT-qPCR and sequencing analysis [21]. 

In the present study, we describe clinical cases registered in 2020–2021, in which SHUV was identified in collected tissue or blood samples. Additionally, we genetically analyze a novel Israeli strain, which was not identified before in Israel. 

## 2. Materials and Methods

### 2.1. Field Samples

Samples from aborted or malformed domestic or wild ruminant fetuses and dead newborn animals comprised placenta, brain, and internal organs; blood samples from abnormal newborns, sick young or adult animals, and females after abortion (whole blood, plasma and serum samples); brains samples from abruptly dead or euthanized animals that manifested neural signs before their death were submitted in 2020–2021 for routine examination to the virology department of Kimron Veterinary Institute (KVI), Israel. Details on the number per year, sample matrix, and animal species from which samples were collected are summarized in Table 1.

### 2.2. Viral RNA/DNA Extraction

For RNA extraction, tissue samples were homogenized in phosphate-buffered saline (PBS) in the proportion 1:10 and centrifuged. Supernatant from tissue culture homogenates was used for RNA extraction. Viral RNA was extracted from all kinds of field samples using the MagMAX™ CORE Nucleic Acid Purification Kit (Thermo Fisher Scientific, Austin, TX, USA), according to the manufacturer’s recommendations.

### 2.3. Laboratory Tests

A generic real-time RT-PCR was applied for the detection of simbuviruses [22]. For the differential diagnosis of viral pathogens in aborted fetuses, the EHDV Real-Time PCR Kit (Applied Biosystems, Thermo Fisher Scientific Inc., Lissieu, France) for detection of epizootic hemorrhagic disease virus (EHDV) was used according to the manufacturer’s instructions. For bluetongue virus (BTV) detection, the VetMAX™ BTV NS3 All Genotypes Kit (Applied Biosystems™, Thermo Fisher Scientific Inc., Lissieu, France) was applied or used an alternative method described by Wernike et al. [23]. For the detection of pestiviruses, the method developed by Wernike et al. was used [23]. For the differential diagnosis of viral pathogens that could possibly cause the appearance of neural signs, animal brains were firstly tested for rabies using a direct immunofluorescence antibody (dIFA) [24] (Rabies laboratory, KVI). Thereafter, the brain samples were tested for malignant catarrhal fever virus (MSFV) [25] and for bovine spongiform encephalopathy (BSE) using the commercially available enzyme-linked immunosorbent assay (ELISA) TeSeE™ SAP Combi Kit (BIO-RAD, Hercules, CA, USA). For differential diagnoses, blood samples from cattle were tested for BTV, EHDV, and bovine ephemeral fever virus (BEFV) [26].

### 2.4. Sequencing and Phylogenetic Analyses

For diagnostic purposes for identification of simbuviruses, a partial sequence of the S segment was applied (panSimbu RT-qPCR) [22]. Primers used for partial sequencing of all three viral segments of SHUV have been previously published [7,27]. For all conventional RT-PCRs, the One-Step RT-PCR kit (Qiagen, Hilden, Germany) was used. The cDNA fragments of positive samples were purified with the MEGAquick-spin Total Fragment DNA Purification Kit (iNtRON Biotechnology, Seongnam-si, Gyeonggi-do, Korea) and subsequently sequenced using standard Sanger methods in both directions using an ABI 3730xl DNA Analyzer (Hylabs, Rehovot, Israel). The resulting nucleotide (nt) sequences were assembled, and nt sequences were aligned and pairwise compared by using Geneious version 9.0.5 (Biomatters, Auckland, New Zealand). Phylogenetic trees were constructed using the Mega X software [28]. For all trees, the maximum-likelihood method was used, and the Tamura–Nei model was applied.

For full-genome sequencing of the SHUV ISR-222/20 and ISR-3024/21 bovine brain samples, RNA was extracted using the Invisorb Spin Virus RNA Mini Kit (STRATEC Molecular, Berlin, Germany) with DNase I (A&A Biotechnology, Gdynia, Poland) treatment before the second washing step.

RNA sequencing libraries were prepared with Illumina-compatible NEBNext^®^ Ultra™ II Directional RNA Library Prep Kit (New England BioLabs, Ipswich, MA, USA) at Genotypic Technology Pvt. Ltd., Bangalore, India. Briefly, 50–500 ng of total RNA was taken for rRNA depletion using a Ribo-minus rRNA Removal Kit (Invitrogen Ribominus Eukayote module). Then, ~50 ng of Qubit quantified ribo-depleted RNA was taken for fragmentation and priming. The fragmented and primed mRNA was further subjected to first-strand synthesis followed by second-strand synthesis. The double-stranded cDNA was purified with NEBNext sample purification beads. Purified cDNA was end-repaired, adenylated, and ligated to Illumina multiplex barcode adapters as per NEBNext^®^ Ultra™ II Directional RNA Library Prep protocol followed by second-strand excision using the USER enzyme at 37 °C for 15 min.

The adapters used in the study were Illumina Universal Adapter.

(5′-AATGATACGGCGACCACCGAGATCTACACTCTTTCCCTACACGACGCTCTTCCGATCT-3′) and Index Adapter (5′-GATCGGAAGAGCACACGTCTGAACTCCAGTCAC [INDEX] ATCTCGTATGCCGTCTTCTGCTTG-3′, where [INDEX] is a unique sequence to identify sample-specific sequencing data).

Adapter-ligated cDNA was purified using JetSeq magnetic beads and was subjected to 10–12 cycles for Indexing (98 °C for 30 s, cycling (98 °C for 10 s, 65 °C for 75 s), and 65 °C for 5 min) to enrich the adapter-ligated fragments. The final PCR product (sequencing library) was purified with NEBNext sample purification beads, followed by a library quality-control check. The Illumina-compatible sequencing library was quantified using a Qubit fluorometer (Thermo Fisher Scientific, Waltham, MA, USA) for its fragment size distribution analysis on Agilent 2200 TapeStation. The mean fragment size of fragments was 355 bp. The libraries were sequenced on an Illumina NovaSeq 6000 sequencer (Illumina, San Diego, CA, USA) using 150 bp paired-end chemistry.

## 3. Results

### 3.1. Clinical Cases and Laboratory Diagnosis

#### 3.1.1. Cattle Abortions

**Figure 1:**
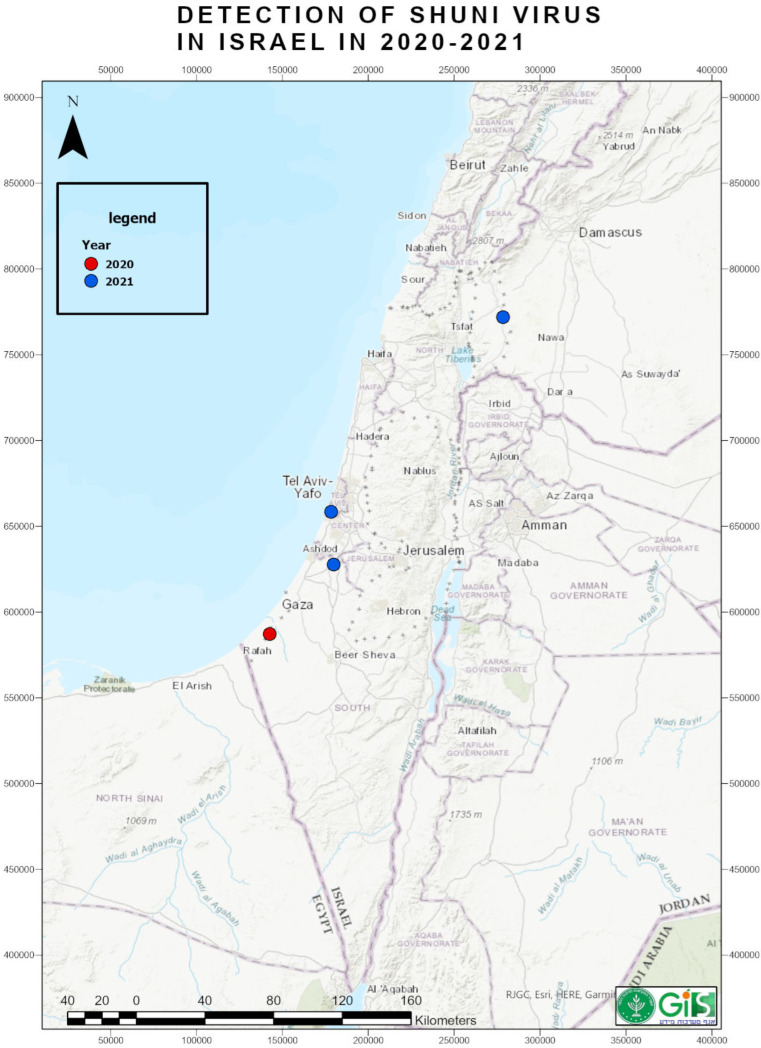
Map of geographic places where Shuni virus infections of domestic ruminants were registered, 2020–2021.

Two aborted fetuses without gross pathological changes were received at KVI on 22 November and 7 December 2020, from the cattle farm in Kissufim, which is located in the northwestern Negev in southern Israel, close to the Gaza Strip (Figure 1). Brain samples from these fetuses were positive in panSimbu RT-qPCR (Cq 14.18 and 26.68, respectively). Tissue samples from the submitted aborted fetuses were tested for BTV and pestiviruses and found negative. The consequent sequence analysis of the S fragment illustrated SHUV. Notably, on 13 December 2020, an aborted cattle fetus (gross pathology examination revealed hydranencephaly) collected from the same farm was AKAV positive. Therefore, the farm was simultaneously affected by AKAV and SHUV in 2020.

#### 3.1.2. Beef Cattle

Three whole-blood samples from 8-year-old beef grazing cows were sent on 25 July, 2021, from the northeast of Golan Heights, near the Bashanit ridge, in the northern part of Israel (Figure 1). All three cows manifested fever (>39 °C), fatigue, encrustation and a dry muzzle, and sharp decrease in the body weight. These cows were treated with antibiotics without improvement of their condition. Blood samples were tested for BTV, EHDV, and BEFV and found negative. One of three samples was positive (Cq 25.88) in panSimbu RT-qPCR, and sequencing analysis of the diagnostic region showed SHUV.

#### 3.1.3. Fattening Calf

On 27 September 2021, a brain from a 10-month-old fattening calf, which manifested neural signs before its death, was sent from a cattle farm located at Yinon, in the southern part of Israel (Figure 1). The brain was tested for rabies virus and MCFV and found negative. The panSimbu RT-qPCR test showed Cq 25.2, and sequencing analysis of diagnostic region revealed SHUV. No additional laboratory findings were identified.

#### 3.1.4. Goat Abortion

A dead newborn kid was collected on 1 November 2021, after delivery by an 11-month-old goat. This goat was kept in the petting zoo, located in Holon, central coastal area of Israel (Figure 1). During the same period, an additional two goats aborted dead fetuses at the end of gestation, but their aborted material was not submitted for laboratory diagnosis to KVI. Macroscopic pathological investigation revealed hydrocephalus of the submitted newborn dead kid. The spleen and lung samples were tested for pestiviruses and BTV and found negative. The brain sample tested in PanSimbu RT-qPCR showed a weak positive result (Cq 37.48), and sequencing of the S segment fragment revealed SHUV.

### 3.2. Total PanSimbu RT-qPCR and Identification of Simbuviruses from Field Samples

In total, 66 out of 932 field samples were positive in panSimbu RT-qPCR. The full data on samples and type of animals are presented in Table 1. In general, only two species of simbuviruses were identified in the submitted samples in 2020–2021. The absolute majority (58) of positive samples were identified as AKAV positive (2018-year-like strains), 5 samples were SHUV positive, and 3 positive samples with a low concentration of viral RNA stayed untyped.

### 3.3. Sequencing and Pairwise and Phylogenetic Analyses

Sequences obtained in this study were uploaded to the GenBank under the accession numbers (ac.num.) ON920928-ON920934 and OP131183-OP131184. Data on ac. num., length of the sequences, and their usage in phylogenetic analyses are present in Table S1.

The complete coding regions of all three SHUV genome segments were sequenced from the brain sample of the cattle aborted fetus (ISR-222/20). M and S segments from the clinical case of the calf with neural signs (ISR-3024/21) and M and L segments from the symptomatic beef cow (ISR-1821/2/21) were partially sequenced and used for phylogenetic analyses. Due to short nt fragment sequences of the S segment used for the identification of simbuviral species, several SHUV S segment sequences from the field strains collected in 2019–2021 were not used in phylogenetic analyses and were not submitted to the GenBank, but they were used in nt and amino acid (aa) alignments (Figures S1 and S2). A completely sequenced ISR-1837/18 strain from the brain of a calf that manifested neural signs before its death (and eventually revealed a mixed brain tissue infection with untyped BTV, ac. num. of SHUV ON087706-ON087708) was also used in all nt and aa sequence analyses. Due to the very close relationship of Israeli strains identified in 2014–2019, three representative strains were taken for phylogenetic analyses.

#### 3.3.1. M Segment Analyses

**Figure 2:**
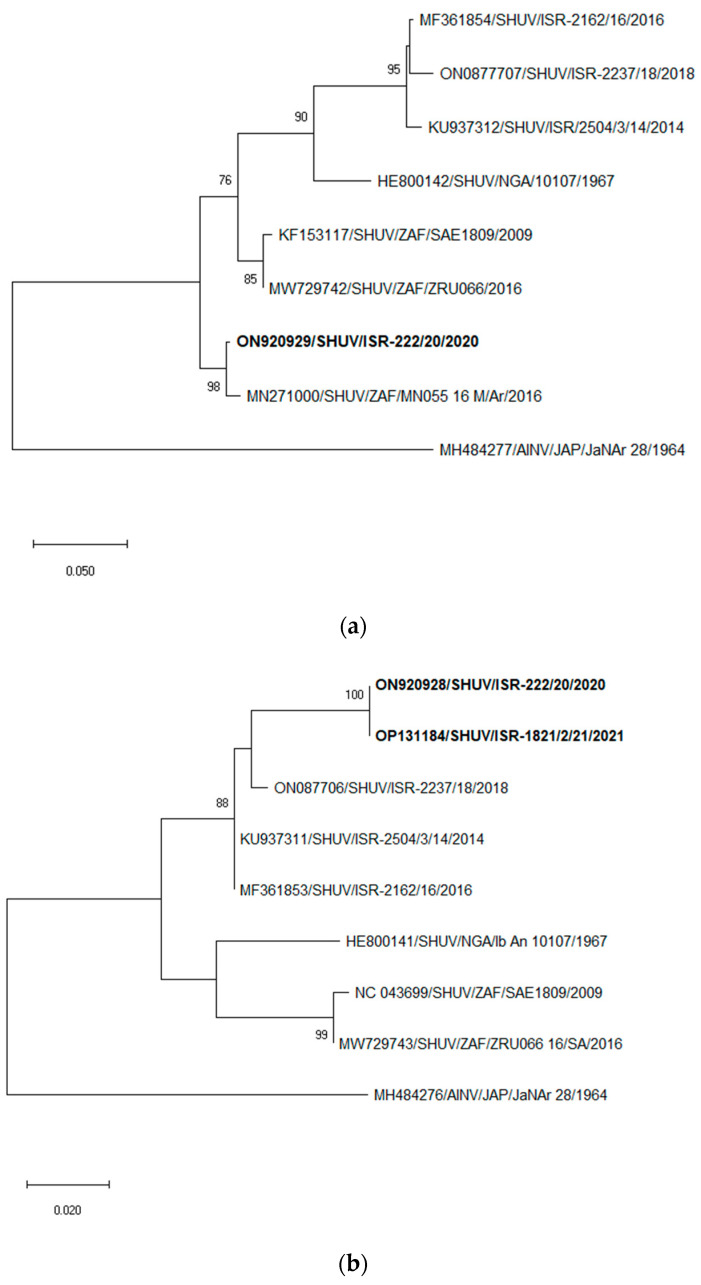

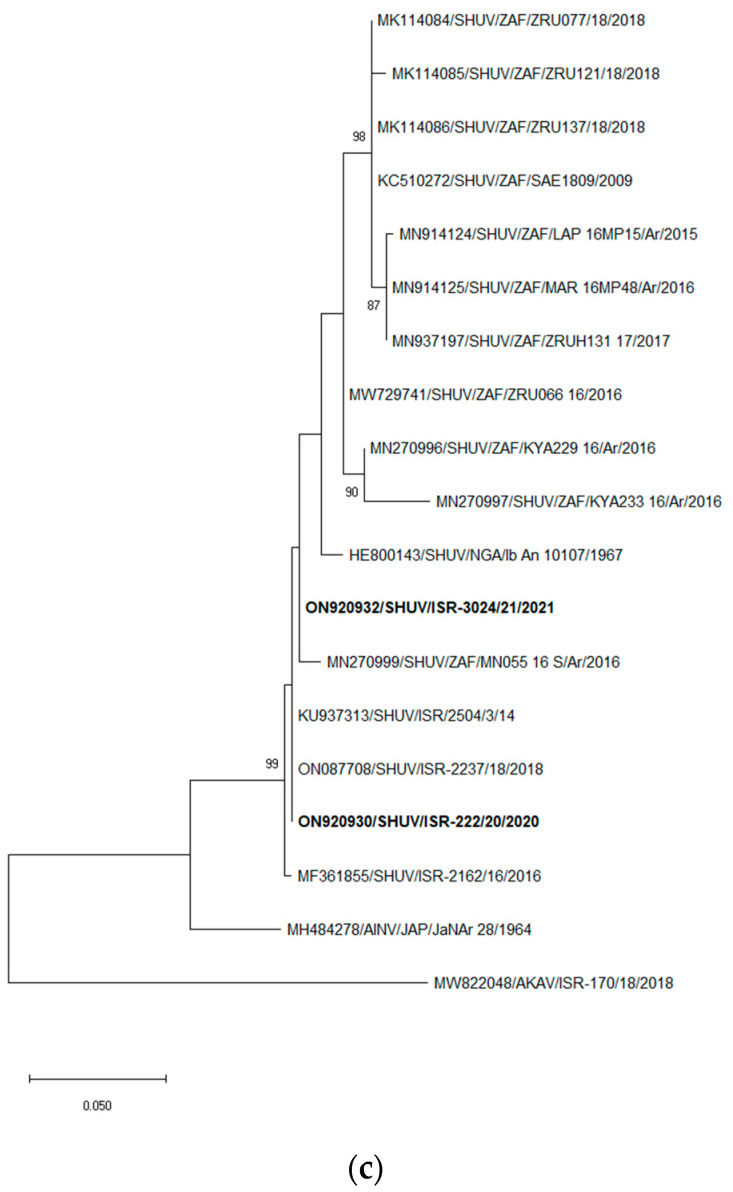
Phylogenetic trees of nucleotide sequences of Israeli and global SHUV. (**a**) M segment sequences; (**b**) L segment sequences; (**c**) S segment sequences. Sequences obtained in this study are in bold. Sequences obtained from the NCBI GenBank are labeled by accession number/viral species/location/isolate/year. Aino virus (AINV) was used as an outgroup for phylogenetic analyses of M and L segments, and Akabane (AKAV) virus was used as an outgroup for phylogenetic analysis of S segment sequences. Nucleotide sequences were analyzed using the maximum-likelihood method and the Tamura–Nei model. Statistical support for nodes was obtained by bootstrapping (1000 replicates); only values ≥70% are shown. Scale bars indicate nucleotide substitutions per site.

Simultaneous comparison of all three SHUV M segment sequences from novel Israeli strains was impossible because of the absence of overlapping sequenced regions of ISR-1821/2/21 and ISR-3024/21 strains, and they were analyzed separately. Pairwise analysis of the same sequenced regions of the M segment of SHUV ISR-222/20 and ISR-1821/2/21 showed 100% identity (751-nt-long compared region), and comparison of ISR-222/20 and ISR-3024/21 showed two silent nt substitutions (538-nt-long compared region). These results allow us to consider these sequences as belonging to very closely related SHUV strains. Pairwise analysis of the M segment of completely sequenced Israeli strains showed that the ISR-222/20 strain had only 89.51–89.66% nt identity with Israeli strains collected during 2014–2018, while the aa sequences of polyprotein showed 96.51–97.08% identity (44–47 aa substitutions). The pairwise analysis with South African SAE1809 and ZRU066_16 strains showed 94.85% and 94.50% nt identity, respectively, and 97.58–97.86% aa identity (29 and 33 aa substitutions, respectively). Pairwise analysis with Nigerian Ib An 10107 showed 91.98% nt and 97.86% aa identity (30 aa substitutions). Pairwise analysis of the Israeli ISR-222/21 strain with the South African MN055_16_M/Ar strain (395 nt) showed 98.74% nt and 100% aa identity. Due to the absence of overlapping regions of South African strain MN055_16_M/Ar with partially sequenced Israeli ISR-1821/2/21 and ISR-3024/21 strains, only the completely sequenced ISR-222/20 strain was used for phylogenetic analysis of the M segment. Phylogenetic analysis of the nt sequences showed that novel Israeli strains by the M segment clustered with the South African strain MN055_16_M/Ar of *Culicoides* spp. origin collected in 2016 (Figure 2a).

#### 3.3.2. L Segment Analyses

Pairwise analysis of the same sequenced regions of the L segment of SHUV ISR-222/20 and ISR-1821/2/21 showed 100% identity (248-nt-long compared regions had a unique sequence compared to all other available SHUV sequences). Pairwise analysis of the L segment of completely sequenced Israeli strains showed that the ISR-222/20 strain had only 96.34–96.90% nt identity with Israeli strains collected during 2014–2018, while BLAST analysis with global non-Israeli strains showed only 92.20–92.39% nt identity with African strains. Pairwise analysis of RdRp protein aa sequences showed 98.76–98.85% identity (24–28 aa substitutions) with previously identified Israeli SHUV strains, while comparison with African strains showed 98.14–98.22% aa identity (40–42 aa substitutions). Phylogenetic analysis of the nt sequences showed that novel Israeli strains by the L segment clustered with the Israeli strains collected a few years before (Figure 2b).

#### 3.3.3. S Segment Analyses

Pairwise analysis of the Israeli ISR-222/21 and ISR-3024/21 common sequences of the S segment region (707 nt) showed 99.58% nt identity and 100% N and NSs proteins identity. Pairwise analysis of the S segment of completely sequenced Israeli strains showed that the ISR-222/20 strain had 99.23% nt identity with both the Israeli 2504/3/14 and ISR-2162/16 strains, whereas, with the ISR-1537/18 Israeli strain, it had 99.52% nt identity. Comparison of the N proteins of all Israeli strains from 2014 to 2021 showed 100% aa identity. BLAST analysis with global, completely sequenced non-Israeli strains showed only 97.58% nt identity with Nigerian Ib An 10107 strain, 96.86% and 97.78% nt identity with the South African SAE1809 and ZRU066_16 strains, respectively. Comparison of the N proteins of Israeli and all African completely sequenced strains showed 100% aa identity. Notably, since 2018, the S segments of Israeli SHUVs sampled in 2018–2019 were specific point mutations in nt sequences at positions 173 and 209, whereas the SHUVs with 2020–2021 dates of collection had only one point mutation at position 173 (Figure S1). Comparing the aa sequences of NSs proteins, it was seen that the Israeli strains with 2018 and 2019 years of collection had substitutions at position T38I (threonine instead of isoleucine) and H50R (histidine instead of arginine), whereas, since 2020, only the H50R substitution has been present, similar to the case with all African SHUV strains (Figure S2). Based on this sequence analysis of the S segment, we believe that the “goat” sample (ISR-190/21) also belonged to the same strain, as all other sequenced SHUV-positive field samples identified in 2020–2021. Partial low-quality S segment sequences of South African strains were also used for phylogenetic analysis in addition to completely sequenced non-Israeli strains. This analysis cannot pinpoint the origin of this segment of the Israeli strains identified in 2020–2021, which illustrated clustering in a single group with all previously identified Israeli SHUV strains and South African MN055_16_M/Ar strain of *Culicoides* spp. origin collected in 2016 (Figure 2c).

In general, the sequence and genetic analyses of M and L segments of Israeli SHUVs collected in 2020–2021 showed a considerably low nt and aa identity with all known SHUV strains, which indicates that the Israeli strain is a novel type of SHUV. At the same time, novel Israeli strains showed about 98–99% nt identity by M and S segment nt sequences with some South African strains collected in 2016–2018, illustrating the probable presence of closely related South African SHUV to recently identified Israeli strains. Different genetic analyses of the S segment showed more that 99% nt identity with all known Israeli strains and probable close relationship and clustering with the “Israeli-like” MN055_16_S/Ar South African strain. More than 99% nt identity of the S segment sequences of all “old” and “new” Israeli SHUVs allows us to presume that the novel Israeli and probably South African MN055_16_S/Ar strains had a reassorted origin.

## 4. Discussion

SHUVs have been circulating in Israel at least since 2014. During 2014–2019, the Israeli SHUV belonged to the same circulated strain, differing in point mutations only. Since 2020, the novel SHUV has been identified, whereas a previously circulated strain has not been detected. The novel strain was found in every geographic region of Israel: in northern, central, and southern parts of Israel. As with the previously circulated Israeli SHUV strains, the novel strains caused all three kinds of clinical manifestation of the disease: abortions in domestic ruminants, neural signs in young cattle, and clinical manifestation in beef cattle. A single confirmed case of SHUV infection of a beef cow with serious clinical signs, when fever (>39 °C), fatigue, encrustation and a dry muzzle, and sharp decrease in the body weight was registered, which does not allow us to determine whether this SHUV strain causes a more harmful acute disease in cattle or not, comparing to described acute clinical cases caused by SBV and SANV infection in milking cows [4,6].

The strong difference of M (less than 92% nt identity with the closest available SHUV strain) and L (96.34–96.90% nt identity with closest SHUV strain) segments from all other knows SHUV strains, whereas only S segments had more than 99% nt identity with previously circulated in 2014–2019 Israeli strains, allows us to deduce a reassortment origin of the strain. Continuous genetic monitoring of Israeli SHUV has revealed a significant difference of novel strains in M and L segments from the previously circulated Israeli SHUVs. On the contrary, analyses of the S segment showed more that 99% nt identity with the Israeli strains and probable close relationship and clustering with the Israeli-like MN055_16_S/Ar South African strain. Moreover, phylogenetic analysis of the S segment of the available South African strains showed the presence and circulation of several genetically different strains in South Africa. These conclusions were indirectly confirmed in publication made by Steyn et al. [18], whereas the presented S segment phylogenetic tree showed several different South African SHUV clusters. Nevertheless, this publication illustrated the clustering of the ZRU087_18 South African strain collected in giraffe in 2018 with Israeli strains collected in 2014–2016. Unfortunately, this sequence is not publicly available, which does not allow S segment comparison with the Israeli novel and “old” strains. A single partial M segment sequence of the MN055_16_S/Ar South African strain also showed a close relationship of the strain with recently identified Israeli SHUV strains. The high identity of S and M segments (about 98–99% nt identity) of the MN055_16_S/Ar South African strain indicates the probable presence of closely related SHUVs to the recently identified Israeli SHUV. These data allow us to presume that the closely related Israeli SHUV strains (not clear novel or also “old” strains) have also been circulating in South Africa. However, a total absence of L segment sequences of recent South African SHUV strains complicates identification of the place and time of appearance of this novel strain and presumes the direction of its spread to a new territory. In case the virus appeared not in Israel, but in South Africa, and was first detected in 2016 (which at the moment is impossible either to prove or to reject), and based on continuous genetic monitoring of Israeli SHUV, we can presume that it is possible that a few years are needed for every arboviral strain to cross the African continent and reach Eurasia.

Due to the fact that this virus was identified in a large range of wild and domestic animals [10,17,18,19] and in a human with a febrile disease [20] and with neural signs of unknown etiology [21], more serious monitoring and surveillance programs are required in preparation for the probable spread of these viruses both in animals and in humans at new territories. 

## Figures and Tables

**Table 1:** Field samples tested by panSimbu RT-qPCR during 2020–2021.

	Aborted Fetus and Newborn							Other Age Categories				
Year		Brain	Placenta	Mixed	Blood	Total Samples	Total Cases	Brain	Internal Organs	Blood	Total Samples	Total
2020	cattle	4/42	1/18	0/1	2/13	7/74	7/65	0/11	0/7	0/240	0/258	7/332
	sheep	18/85	15/56	0/5	0/2	33/148	20/101	0/11	0/1	1/35	1/47	34/195
	goat	4/9	0/4	0	0	4/13	4/10	0/4	0	0/2	0/6	4/19
	wild/zoo	0/6	0/2	0	0	0/8	0/6	0/12	0	0/3	0/15	0/23
	total	26/142	16/80	0/6	2/15	44/243	31/182	0/38	0/8	1/280	1/326	45/569
2021	cattle	2/45	1/16	0/1	0/13	3/75	3/68	1/5	0	1/136	2/141	5/216
	sheep	6/60	6/34	0	0/1	12/95	10/72	0/4	0	0/23	0/27	12/122
	goat	2/7	2/4	0	0	4/11	3/10	0/1	0/1	0/4	0/6	4/17
	wild/zoo	0	0	0	0	0	0	0/8	0	0	0/8	0/8
	total	10/112	9/54	0/1	0/14	19/181	16/150	1/18	0/1	1/163	2/182	21/363
total		36/254	26/134	0/7	2/29	63/424	45/332	1/56	0/9	2/443	3/508	66/932

Wild/zoo: wild or zoo animals; blood: blood samples, including whole blood, plasma, or serum samples. The number of positive results/total number of investigated samples or number of positive abortion cases/total number of tested abortion cases are shown.

## Data Availability

Not applicable.

## Supplementary Materials

The following supporting information can be downloaded at: https://www.mdpi.com/article/10.3390/tropicalmed7100297/s1, Figure S1: Nucleotide alignment of S segment region of Israeli and global SHUV. Dots showed the same nucleotide in the position; Figure S2: Amino acid alignment of nonstructural S protein of Israeli and global SHUV. X-incomplete codon of the amino acid. Dots showed the same amino acid in the position; Table S1: Information on Israeli Shuni virus strains collected in 2020–2021.

## References

[1] Sick F., Beer M., Kampen H., Wernike K. (2019). Culicoides Biting Midges-Underestimated Vectors for Arboviruses of Public Health and Veterinary Importance. Viruses.

[2] Elliott R.M. (2014). Orthobunyaviruses: Recent genetic and structural insights. Nat. Rev. Microbiol..

[3] Plyusnin A., Elliott R.M., Plyusnin A., Elliott R.M. (2011). Bunyaviridae: Molecular and Cellular Biology.

[4] Beer M., Wernike K., Bamford D.H., Zuckerman M. (2021). Akabane virus and Schmallenberg virus (*Peribunyaviridae*). Virology.

[5] Kirkland P.D. (2015). Akabane virus infection. Rev. Sci. Tech..

[6] Golender N., Bumbarov V., Eldar A., Zamir L., Even-Tov B., Gabriel K., Eitan T. (2021). Isolation of Sango viruses from Israeli symptomatic cattle. Int. J. Veter Sci. Res..

[7] Golender N., Bumbarov V., Assis I., Beer M., Khinich Y., Koren O., Edery N., Eldar A., Wernike K. (2019). Shuni virus in Israel: Neurological disease and fatalities in cattle. Transbound. Emerg. Dis..

[8] Sick F., Breithaupt A., Golender N., Bumbarov V., Beer M., Wernike K. (2021). Shuni virus-induced meningoencephalitis after experimental infection of cattle. Transbound. Emerg. Dis..

[9] Kono R., Hirata M., Kaji M., Goto Y., Ikeda S., Yanase T., Kato T., Tanaka S., Tsutsui T., Imada T. (2008). Bovine epizootic encephalomyelitis caused by Akabane virus in southern Japan. BMC Vet. Res..

[10] van Eeden C., Williams J.H., Gerdes T.G., van Wilpe E., Viljoen A., Swanepoel R., Venter M. (2012). Shuni virus as cause of neurologic disease in horses. Emerg. Infect. Dis..

[11] Yanase T., Kato T., Aizawa M., Shuto Y., Shirafuji H., Yamakawa M., Tsuda T. (2012). Genetic reassortment between Sathuperi and Shamonda viruses of the genus *Orthobunyavirus* in nature: Implications for their genetic relationship to Schmallenberg virus. Arch. Virol..

[12] Garigliany M.M., Bayrou C., Kleijnen D., Cassart D., Jolly S., Linden A., Desmecht D. (2012). Schmallenberg virus: A new Shamonda/Sathuperi-like virus on the rise in Europe. Antiviral Res..

[13] Bowen M.D., Trappier S.G., Sanchez A.J., Meyer R.F., Goldsmith C.S., Zaki S.R., Dunster L.M., Peters C.J., Ksiazek T.G., Nichol S.T. (2001). A reassortant bunyavirus isolated from acute hemorrhagic fever cases in Kenya and Somalia. Virology.

[14] Kobayashi T., Yanase T., Yamakawa M., Kato T., Yoshida K., Tsuda T. (2007). Genetic diversity and reassortments among Akabane virus field isolates. Virus Res..

[15] Oymans J., Wichgers Schreur P.J., van Oort S., Vloet R., Venter M., Pijlman G.P., van Oers M.M., Kortekaas J. (2020). Reverse Genetics System for Shuni Virus, an Emerging Orthobunyavirus with Zoonotic Potential. Viruses.

[16] Causey O.R., Kemp G.E., Causey C.E., Lee V.H. (1972). Isolations of Simbu-group viruses in Ibadan, Nigeria 1964–69, including the new types Sango, Shamonda, Sabo and Shuni. Ann. Trop. Med. Parasitol..

[17] Golender N., Brenner J., Valdman M., Khinich Y., Bumbarov V., Panshin A., Edery N., Pismanik S., Behar A. (2015). Malformations Caused by Shuni Virus in Ruminants, Israel, 2014–2015. Emerg. Infect. Dis..

[18] Steyn J., Motlou P., van Eeden C., Pretorius M., Stivaktas V.I., Williams J., Snyman L.P., Buss P.E., Beechler B., Jolles A. (2020). Shuni Virus in Wildlife and Nonequine Domestic Animals, South Africa. Emerg. Infect. Dis..

[19] Golender N., Bumbarov V., Kovtunenko A., David D., Guini-Rubinstein M., Sol A., Beer M., Eldar A., Wernike K. (2021). Identification and Genetic Characterization of Viral Pathogens in Ruminant Gestation Abnormalities, Israel, 2015–2019. Viruses.

[20] Moore D.L., Causey O.R., Carey D.E., Reddy S., Cooke A.R., Akinkugbe F.M., David-West T.S., Kemp G.E. (1975). Arthropod-borne viral infections of man in Nigeria, 1964–1970. Ann. Trop. Med. Parasitol..

[21] Motlou T.P., Venter M. (2021). Shuni Virus in Cases of Neurologic Disease in Humans, South Africa. Emerg. Infect. Dis..

[22] Golender N., Bumbarov V.Y., Erster O., Beer M., Khinich Y., Wernike K. (2018). Development and validation of a universal S-segment-based real-time RT-PCR assay for the detection of Simbu serogroup viruses. J. Virol. Methods.

[23] Wernike K., Hoffmann B., Beer M. (2015). Simultaneous detection of five notifiable viral diseases of cattle by single-tube multiplex real-time RT-PCR. J. Virol. Methods.

[24] Smith J.S., Murray P.R., Baron E.J., Pfaller M.A., Tenover F.C., Yolken R.H. (1995). Rabies virus. Manual of Clinical Microbiology.

[25] Cunha C.W., Otto L., Taus N.S., Knowles D.P., Li H. (2009). Development of a multiplex real-time PCR for detection and differentiation of malignant catarrhal fever viruses in clinical samples. J. Clin. Microbiol..

[26] Erster O., Stram R., Menasherow S., Rubistein-Giuni M., Sharir B., Kchinich E., Stram Y. (2017). High-resolution melting (HRM) for genotyping bovine ephemeral fever virus (BEFV). Virus Res..

[27] Fischer M., Schirrmeier H., Wernike K., Wegelt A., Beer M., Hoffmann B. (2013). Development of a pan-Simbu real-time reverse transcriptase PCR for the detection of Simbu serogroup viruses and comparison with SBV diagnostic PCR systems. Virol. J..

[28] Kumar S., Stecher G., Li M., Knyaz C., Tamura K. (2018). MEGA X: Molecular evolutionary genetics analysis across computing platforms. Mol. Biol. Evol..

