# Supplementary material for: Identification of Novel Reassortant Shuni Virus Strain in Clinical Cases of Israeli Ruminants, 2020–2021
Source: Trop Med Infect Dis. 2022 Oct 13;7(10):297. doi: 10.3390/tropicalmed7100297 (PMC9606876; doi:10.3390/tropicalmed7100297)
Supplement: Supplementary file 1 [file tropicalmed-07-00297-s001.zip › tropicalmed-1917488-supplementary.pdf]

Tropical Medicine and Infectious Disease

MDPI

Supplementary Materials: Identification of Novel Reassortant Shuni Virus Strain in Clin-ical Cases of Israeli Ruminants, 2020–2021

Table S1. Information on Israeli Shuni virus strains collected in 2020–2021.

	S Segment			M Segment			L Segment		
Strain	Ac. Num	Seq. Region	Phyl. Analysis	Ac. Num	Seq. Region	Phyl. Analysis	Ac. Num	Seq. Region	Phyl. Analysis
ISR-222/20	ON920930	14–842/852	yes	ON920929	14–4274/4351	yes	ON920928	27–6886/6910	yes
ISR-3024/21	ON920932	47–753/852	yes	ON920931	656–1193/4351	no	-	-	-
ISR-1821/2/21	-	-	-	OP131184	3574–4323/4351	no	OP131183	2924–3171/6910	yes
ISR-190/21	-	60–275/852	no	-	-	-	-	-	-

Ac. Num- accession number; Seq. Region- sequenced region/total length of the segment sequence; Phyl. Analysis- phylogenetic analysis.

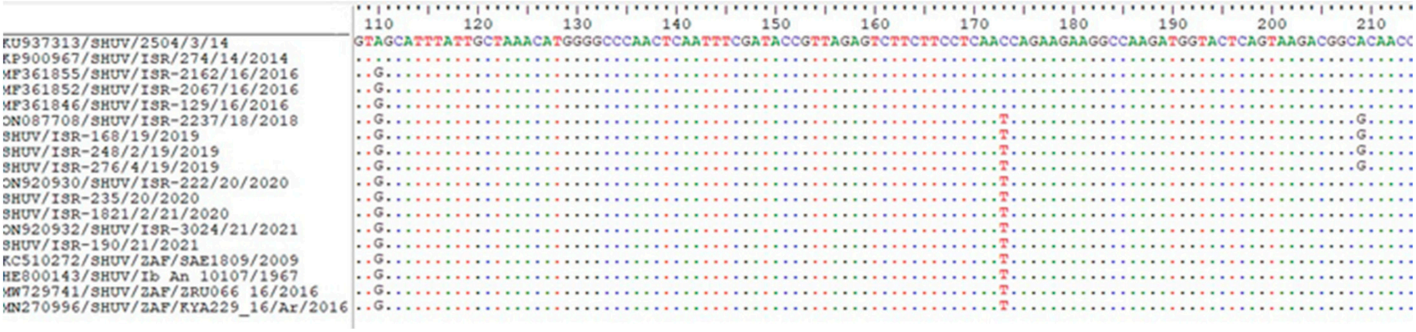

Figure S1. Nucleotide alignment of S segment region of Israeli and global SHUV. Dots showed the same nucleotide in the position.

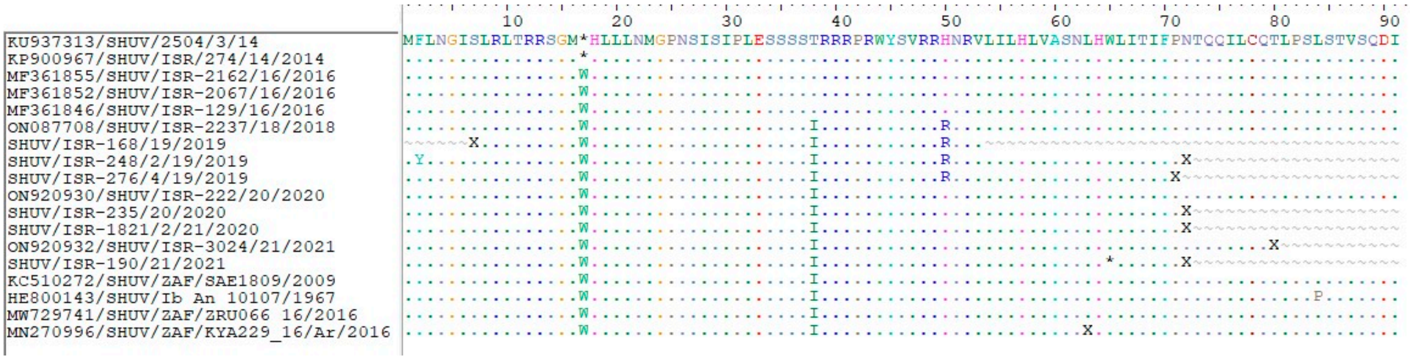

Figure S2. Amino acid alignment of nonstructural S protein of Israeli and global SHUV. X- incom-plete codon of the amino acid. Dots showed the same amino acid in the position.
